# The Effect of Natural Feline Coronavirus Infection on the Host Immune Response: A Whole-Transcriptome Analysis of the Mesenteric Lymph Nodes in Cats with and without Feline Infectious Peritonitis

**DOI:** 10.3390/pathogens9070524

**Published:** 2020-06-29

**Authors:** Alexandra J. Malbon, Giancarlo Russo, Carole Burgener, Emi N. Barker, Marina L. Meli, Séverine Tasker, Anja Kipar

**Affiliations:** 1 Institute of Veterinary Pathology, Vetsuisse Faculty, University of Zurich, CH 8057 Zürich, Switzerland; carole.burgener@uzh.ch (C.B.); anja.kipar@uzh.ch (A.K.); 2 Centre for Clinical Studies, Vetsuisse Faculty, University of Zurich, CH 8057 Zürich, Switzerland; mmeli@vetclinics.uzh.ch; 3 Functional Genomics Center Zurich, University of Zurich, CH 8057 Zürich, Switzerland; giancarlo.russo@fgcz.ethz.ch; 4 Langford Clinical Veterinary Service, University of Bristol, Langford, Bristol BS40 5DU, UK; emi.barker@bristol.ac.uk; 5 Bristol Veterinary School, University of Bristol, Langford, Bristol BS40 5DU, UK; s.tasker@bristol.ac.uk; 6 Clinical Laboratory, Department of Clinical Diagnostics and Services, Vetsuisse Faculty, University of Zurich, CH 8057 Zürich, Switzerland; 7 The Linnaeus Group, Shirley, Solihull B90 1BN, UK

**Keywords:** RNA-seq, next-generation sequencing, cell mediated immunity, humoral immunity, FCoV, FIP

## Abstract

Feline infectious peritonitis (FIP) is a coronavirus-induced disease of cats, in which the immune system is known to play a crucial, but complex, role in the pathogenesis. This role is still incompletely understood, with involvement of both host and viral factors. To evaluate differential gene expression and pathway involvement in feline coronavirus (FCoV) infection and FIP, we applied next-generation RNA-sequencing of the mesenteric lymph nodes from cats with naturally-acquired FIP, as well as those with systemic FCoV infection without FIP, and those with neither. Viral infection was associated with upregulation of viral defenses regardless of the disease state, but to a greater degree in FIP. FIP was associated with higher pro-inflammatory pathway enrichment, whilst non-FIP FCoV-positive cats showed lower enrichment of humoral immunity pathways, below that of uninfected cats in the case of immunoglobulin production pathways. This host response is presumed to be protective. In FIP, downregulation of T cell-related processes was observed, which did not occur in non-FIP FCoV-positive cats. These results emphasize the importance of the host’s immune balance in determining the outcome of the FCoV infection.

## 1. Introduction

Feline infectious peritonitis (FIP) is a fatal immune-mediated disease of domestic and wild felids caused by feline coronavirus (FCoV), a highly prevalent virus with a worldwide distribution which infects domestic and wild felids. FCoV is an alphacoronavirus, similar to several respiratory and enteric pathogens in other species, such as canine coronavirus (CoV), porcine transmissible gastroenteritis CoV, and the ‘common cold’ CoVs of humans. It differs at the genus level from the betacoronaviruses that include the severe respiratory disease-causing CoVs of humans (SARS-CoV 1 and 2, MERS-CoV), and the gammacoronaviruses of birds. As a member of the subfamily *Orthocoronavirinae*, it belongs to a different subfamily than known nidoviruses of reptiles and mammals [1,2]. FCoV initially infects enterocytes via the fecal-oral route and in most cases causes only a mild or subclinical enteric disease. The first stage of gaining increased pathogenicity involves infecting monocytes, enabling systemic spread [3]; this may occur in cats which remain healthy without progression to FIP [4]. In ~5% of infected cats, a combination of, as yet only partially understood viral and host factors lead to the development of FIP [5,6]. FIP is characterized by (pyo) granulomatous phlebitis, generally accompanied by more extensive pyogranulomatous lesions and, in many cases, cavitary effusions [7,8,9].

Numerous studies have tried to pinpoint a key viral mutation leading to the disease of FIP. However, this has so far proven inconclusive [10,11]. Certain amino acid switches in the spike protein (which mediates host binding) were initially linked to pathogenicity but have since been shown to indicate systemic spread [12,13,14,15]. The quest for a defining mutation is also made more difficult by the large size of the virus (30 Mb), and its high mutation rate. The latter is a consequence of the low fidelity of the viral RNA polymerase and high tendency for natural homologous recombination due to random template switching during the complex viral replication [16,17,18]. Together, these factors mean that sequencing of the virus coding RNA alone may be insufficient to identify pathogenicity markers.

Susceptibility to disease has been shown to be heritable to some extent, suggesting a genetic component [19,20,21]. Additionally, experimental studies with known pathogenic forms of FCoV have shown that some cats remain resistant to infection, confirming the importance of the host response in disease susceptibility [22,23,24]. Therefore, the immune response of healthy infected cats is of particular interest. Disentangling the virus and host effect in natural infections is complex; given the current absence of clearly defined viral markers of pathogenicity, it cannot be definitively determined whether healthy systemically FCoV-infected cats are those who are resistant, through suppressing a high viral replication as well as limiting their own inflammatory response, or are instead infected with a ‘non-pathogenic’ form. However, as the pathogenic forms arise from mutations to the non-pathogenic form, hosts which are able to suppress viral replication will, in any case, reduce their chances of succumbing to the disease. It is known that systemically infected disease-free cats have lower viral levels than those with FIP [12,25], but also that, experimentally at least, monocytes/macrophages are less supportive of replication of ‘non-pathogenic’ strains [26,27].

The hallmark pathological processes of FIP, monocyte-mediated granulomatous phlebitis and systemic endothelial activation, the changes in the lymphatic tissue (such as lymphocyte depletion) together with clinical signs such as fever, all indicate an excessive and inappropriate immune response [3,28,29]. Therefore, inflammatory cytokines have been a frequent focus of study, yet no single organ has been shown to be convincingly responsible for the apparent cytokine storm [30,31,32,33]. Furthermore, there is evidence that non-primary immune organs such as the liver and heart may also contribute to cytokine production, adding an amplification step [34]. Despite the disease being caused by an excessive immune reaction, immunosuppressed cats are known to be predisposed to FIP [35], suggesting that mounting a successful immune response is a precarious balancing act which we do not yet sufficiently understand. Identifying genetic markers of susceptibility has also been a focus of study; however, genes first found to be of interest in specific breeds subsequently lost significance in more widespread population searches [20,36]. Counter-intuitively, inbreeding of resistant cats led to increased rather than reduced susceptibility [37], supporting the theory that loss of heterozygosity represents the so-called hybrid vigor risk factor. 

Utilizing a rare biobank from cats with FIP, as well as FCoV-infected and uninfected cats without FIP, we previously evaluated mesenteric lymph nodes (MLN) for expression of selected immune mediators and found a possible intermediate stage of activation, represented by upregulation of some mediators, in FCoV-infected cats without FIP [12]. Natural FCoV infection is via the fecal-oral route and the MLN guard the gateway between the external (i.e., intestinal) and internal environment, able to detect and respond first to the inadvertent entry of pathogens [38]. Together with other hemolymphatic organs, MLN have previously been assessed for levels of specific cytokines and for changes in leukocyte populations in FIP [30,39,40,41]. In the current study, we have used next-generation RNA-sequencing of the MLN to evaluate differential gene expression and pathway involvement in FCoV infection and FIP. In the context of FIP, this technique has thus far only been applied in an in vitro study and to peritoneal macrophages of experimentally-infected cats [42,43,44]. In both settings, apoptosis-related genes were found to be highly upregulated, consistent with observations at a morphological level of lymphoid depletion [39,41]. Peritoneal macrophages were not found to show convincing Th2 polarization in FIP [42] despite the common understanding that cell-mediated immunity is protective and Th2 (humoral) immunity is detrimental [45]. Antibody-dependent enhancement is thought by many to be a feature of the disease [6,46,47,48]. However, cross talk between individual cells and cell types is crucial to determining the balance and response type of the immune system. Therefore, we aimed to evaluate the immune response at an organ level in natural infections, which mirror the real-life clinical situation. We hypothesized that cats with FIP would show a higher differential expression of humoral immunity than those with FCoV infection only, as well as an overall higher activation level of pro-inflammatory pathways. We found that, in contrast to cats with FIP (hitherto referred to as FIP cats for brevity), FCoV-infected cats without FIP showed a lower enrichment of humoral immunity pathways (hitherto referred to as non-FIP cats for brevity). Conversely, cats with FIP exhibited downregulation of T cell-related processes, the loss of which can be predicted to impair the immune response. Enhancing our understanding of the host response and genetic determinants of the disease outcome might not only allow for future targeting of therapeutics but potentially also for targeted breeding programs.

## 2. Results

Reverse-transcriptase-quantitative PCR (RT-qPCR) results for FCoV in the MLN of these cases have been previously published [12,13,14] and are included with the signalment in Supplementary Table S1. These showed viral loads to be far higher in FIP cats than in FCoV-positive non-FIP cats, the former having cycle threshold values ranging from 15.1 to 19.8 and the latter from 34.9 to 39.8 (indicating a factor difference of over 10^4^ at the closest values).

### 2.1. Read Quality and Summary of Transcriptional Profiling

The sequencing of the RNA libraries generated on average 25.3 Mio reads (7.1–58.1, SD = 15.1). The mappability of these reads was high (89.7% on average) and remarkably consistent, with a narrow range of 87.4 to 89.8%. Between 59 and 72% of the mapped reads were assigned to coding regions and contributed to the estimation of the gene expression levels. Together with the summary of the quantification and the pair-wise comparisons, this is shown in Supplementary Figure S1. Moreover, the fraction of the ribosomal reads in the samples and their strandedness is shown in Supplementary Figure S2.

**Figure 1:**
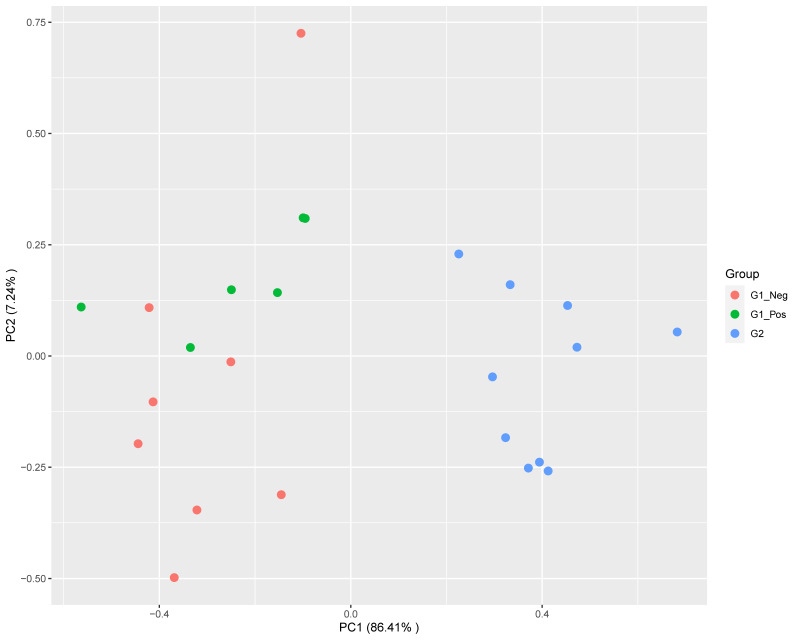
Multidimensional scaling plot showing a main segregation of feline infectious peritonitis (FIP) cats (G2) from both G1 (non-FIP) subgroups (feline coronavirus (FCoV) negative G1_Neg; FCoV positive G1_Pos) along the leading dimension. Segregation within G1 is also observed, but to a lesser extent.

Based on the expression profiles, a clear separation between FIP cats and non-FIP cats was obtained by means of multidimensional scaling (MDS) (Figure 1). To a lesser extent, non-FIP cats also clustered into FCoV-positive and FCoV-negative along the secondary axis. In order to ensure that the effect of secondary variables was not acting as a confounder, we performed additional MDS clustering with additional annotation and a PERMANOVA analysis. Neither secondary data structure nor confounding effects from covariates were found (Supplementary Figure S3). The number of dysregulated genes identified by differential expression (DE) analysis for the pairwise comparisons revealed that differences within the non-FIP group (i.e., between those infected and those not infected with FCoV) were comparatively limited, with approximately 5% the number of DE genes (146) as when the FIP group was compared to either (or a combined) FIP group (G1, G1_Neg, G1_Pos, with 2845, 2565, and 2144 DE genes, respectively). 

### 2.2. FIP Cats Versus Non-FIP Cats (G2 vs. G1)

**Figure 2:**
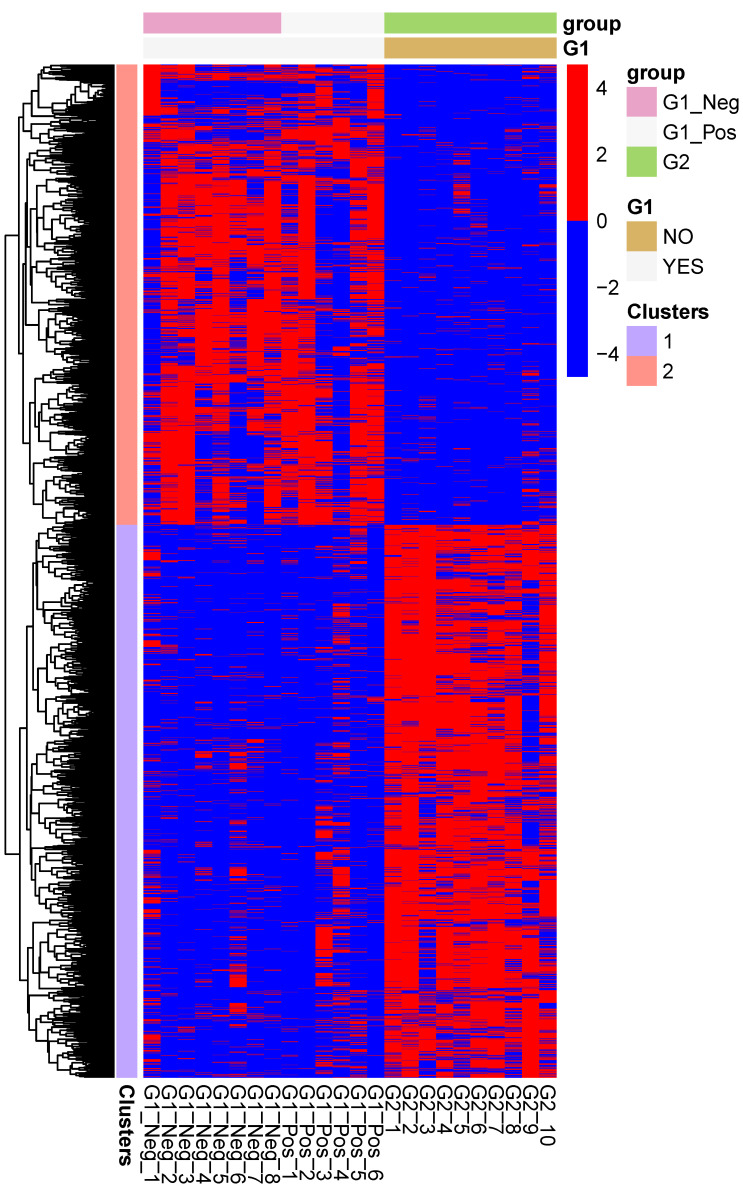
Heatmap of the expression levels of the genes which are differentially regulated between mesenteric lymph nodes from cats with FIP (G2), and cats without FIP (G1_Pos and G1_Neg). The expression levels are log-normalized and scaled row-wise.

Figure 2 shows the expression profiles of the significantly up and downregulated genes from the MLN of FIP cats (G2) as compared to the non-FIP cats (G1). Two well-defined gene clusters are visible, representing transcriptional signatures for FIP and the non-FIP groups.

In order to perform an ensemble functional analysis of the differences in the transcriptional patterns between the groups, we selected from the pair-wise comparisons genes with a *p*-value below 0.05 and a log2ratio above (or below) 0.5. These sets of up or down regulated genes were used as inputs for the GO databases.

**Figure 3:**
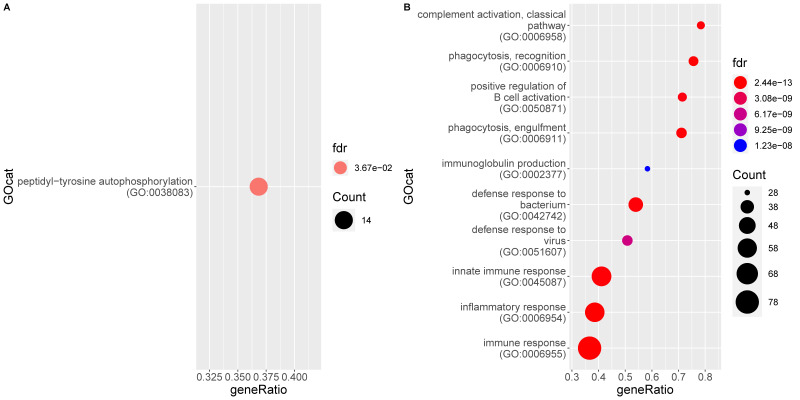
Enrichment of gene ontology (GO) categories based on genes differentially regulated in mesenteric lymph nodes of FIP cats (G2) as compared to the non-FIP cats (G1). The hypergeometric test without replacement is used. “Count” is the number of genes belonging to the category and also differentially expressed in the comparison while the x-axis is the ratio between “Count” and the total number of genes in the category. “Fdr” is the Benjamini-Hochberg-adjusted *p*-value derived from the hypergeometric test. (**A**) Downregulation driven enrichment in FIP; (**B**) upregulation driven enrichment in FIP.

Functional analysis of the genes significantly upregulated in FIP reveals 83 enriched gene ontology (GO) terms associated to a biological process (BP) (Supplementary Table S2). They all refer either directly to the immune system or to a related process (e.g., signaling pathways). The significant processes represent responses to both viral (e.g., defense response to virus) and bacterial (e.g., defense response to bacterium) infection as well as phagocytosis, apoptosis, and pro-inflammatory processes such as IL-6 production, chemotaxis, and complement activation. Both innate and adaptive immune processes are enriched, with multiple terms referring to B cell signaling, activation, and antibody production. Figure 3 shows the ten most enriched GO BP terms of this analysis. Molecular function (MF) terms are similar to BP terms, whilst cellular component (CC) categories mainly relate to the endoplasmic reticulum and Golgi apparatus, presumed secondary to viral packaging [49].

When individual genes are looked at, and ranked by fold-change between groups, the top 20 upregulated in FIP includes IRF7, IFIT2&3, and ISG15 (all IFN pathway genes) as well as C5, CCL8, and CXCL10 (complement pathway component and chemokines, respectively).

A single BP GO term is higher in non-FIP cats: Peptidyl-tyrosine autophosphorylation; however, the CC term GO:0042101 (T cell receptor complex) is also higher in these cats.

### 2.3. FIP Cats Versus Non-FIP Cats Without Detectable FCoV in the Mesenteric Lymph Nodes (G2 vs. G1_Neg)

**Figure 4:**
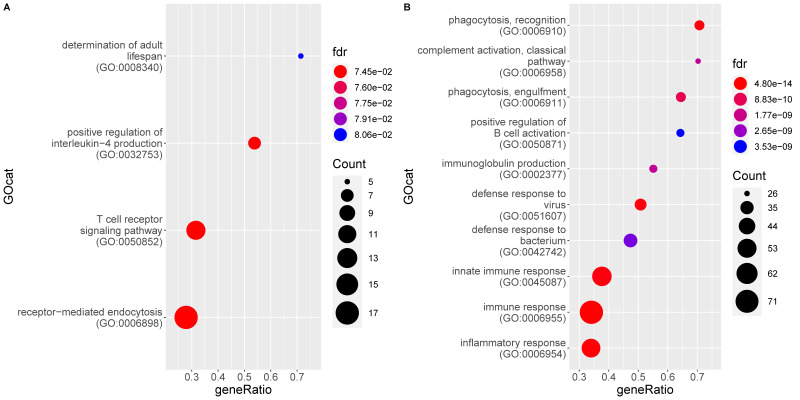
Enrichment of gene ontology (GO) categories based on genes differentially regulated in mesenteric lymph nodes from FIP cats (G2) as compared to FCoV-negative non-FIP cats (G1_Neg). The hypergeometric test without replacement was used. “Count” is the number of genes belonging to the category and also differentially expressed in the comparison while the x-axis is the ratio between “Count” and the total number of genes in the category. “Fdr” is the Benjamini-Hochberg-adjusted *p*-value derived from the hypergeometric test. (**A**) Downregulation driven enrichment in FIP; (**B**) upregulation driven enrichment in FIP.

Figure 4 shows the top BP GO terms enriched for genes significantly up and downregulated in the MLN of FIP cats, respectively, as compared to FCoV-negative non-FIP cats without FIP (G1_Neg). The former consists of 90 GO terms, overlapping heavily with the previous comparison, of MLN from cats with FIP versus cats without FIP (see Supplementary Table S3). Almost all terms enriched in FIP relate directly to the immune response, with some indirectly related, e.g., protein secretion and transport required for processes such as cytokine production. Both innate and acquired immune responses are represented including inflammatory cytokines and immunoglobulin production. Both anti-viral and anti-bacterial pathways are enriched. The top DE genes higher in MLN from FIP cats again include CCL8 and interferon-inducible/related genes. The main differences are in the terms downregulated in MLN from FIP cats which are more numerous in this comparison; there are four terms including immune specific ‘T cell receptor signaling pathway’ and ‘positive regulation of IL-4 production’. 

### 2.4. FIP Cats Versus Non-FIP Cats with FCoV Positive Mesenteric Lymph Nodes (G2 vs. G1_Pos)

**Figure 5:**
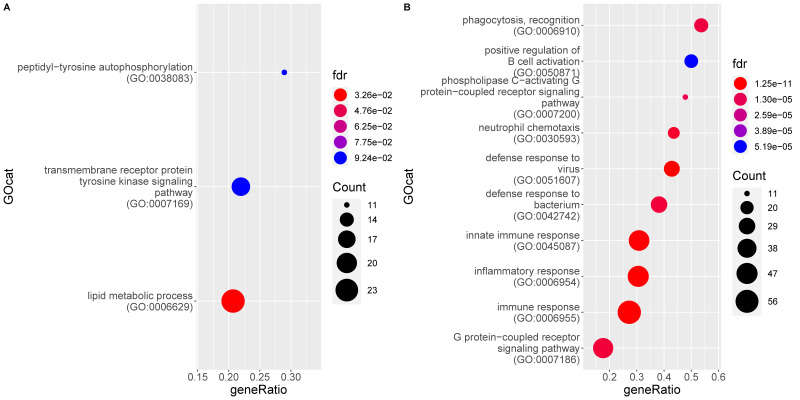
Enrichment of gene ontology (GO) categories based on genes differentially regulated in mesenteric lymph nodes from FIP cats (G2) as compared to FCoV-positive non-FIP cats (G1_Pos). The hypergeometric test without replacement was used. “Count” is the number of genes belonging to the category and also differentially expressed in the comparison while the x-axis is the ratio between “Count” and the total number of genes in the category. “Fdr” is the Benjamini-Hochberg-adjusted *p*-value derived from the hypergeometric test. (**A**) Downregulation driven enrichment in FIP; (**B**) upregulation driven enrichment in FIP.

The functional analysis of differentially expressed genes between these groups reveals that the significantly enriched GO terms are again almost exclusively driven by genes upregulated in the MLN of FIP cats, with three exceptions (Figure 5). The most enriched terms associated to genes upregulated in FIP are again highly similar to the previous pairwise comparisons and include viral defense responses and phagocytosis (see Supplementary Table S4). Individual genes higher in FIP are similar to those listed above.

### 2.5. Non-FIP Cats with and without Detectable FCoV in the MLN (G1_Pos vs. G1_Neg)

**Figure 6:**
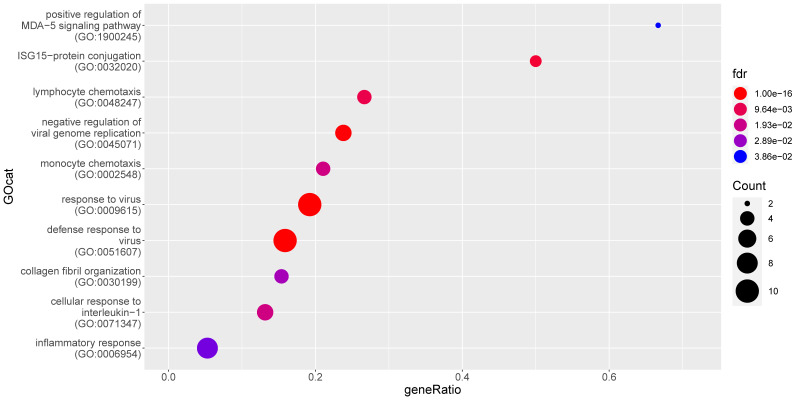
Enrichment of gene ontology (GO) categories based on genes differentially upregulated in mesenteric lymph nodes from FCoV-positive non-FIP cats (G1_Pos) as compared to FCoV-negative non-FIP cats (G1_Neg). The hypergeometric test without replacement was used. “Count” is the number of genes belonging to the category and also differentially expressed in the comparison while the x-axis is the ratio between “Count” and the total number of genes in the category. “Fdr” is the Benjamini-Hochberg-adjusted *p*-value derived from the hypergeometric test.

Comparing the MLN data from the subgroups of non-FIP cats (i.e., FCoV-positive G1_Pos vs. FCoV-negative G1_Neg) resulted in far fewer DE genes and hence fewer significantly enriched GO terms (15; see Figure 6 and Supplementary Table S5, all of which were enriched up in the FCoV infection). Terms higher in FCoV positive cats (G1_Pos) relate to viral defense, leukocyte chemotaxis, and cytokine/chemokine signaling and responses. Bacterial defense and phagocytosis are not observed to be enriched. Many IFN related genes are higher in FCoV positive cats (G1_Pos).

### 2.6. Overlap Comparison of the Host Response to FCoV in FIP Cats and Non-FIP Cats (G2 vs. G1_Neg and G1_Pos vs. G1_Neg)

**Figure 7:**
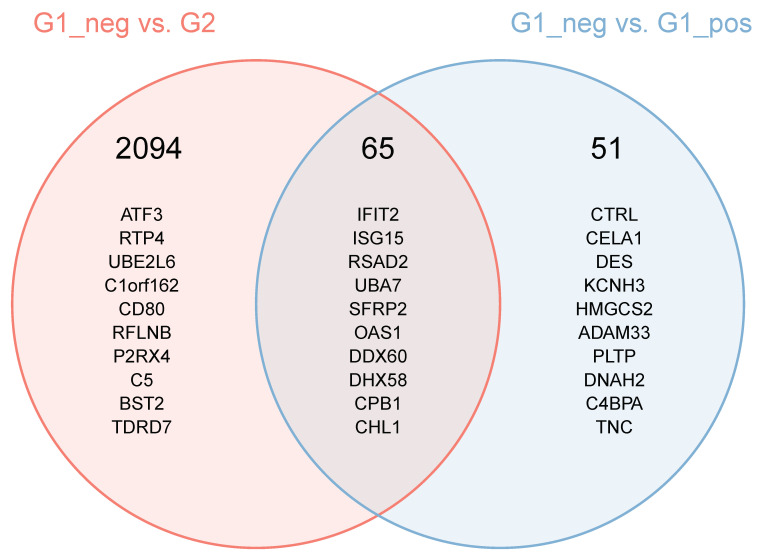
Intersection of the set of differentially expressed genes in the comparisons between FIP cats and non-FIP cats with and without detectable levels of FCoV. The header number represents the number of genes found in the areas of the Venn diagram. The genes listed are the 10 most differentially expressed in the comparisons. For the genes which are differentially expressed in both comparisons, the ranking is based on a score calculated as follows (the lower the score, the higher the ranking): Score = [*p* (comp1) + *p* (comp2)]/abs (log2Ratio (comp1) + log2Ration (comp2)).

Results of the two comparisons between FCoV-positive and FCoV-negative cats (G2 vs. G1_Neg and G1_Pos vs. G1_Neg) were themselves compared to identify the extent of differences in the MLN response when infection outcome (i.e., FIP or not) was considered. A Venn diagram (Figure 7) shows that of all significant DE genes in the two comparisons, 2094 are significant only in the FIP comparison (G2 vs. G1_Neg), 51 only in the comparison between FCoV-positive and FCoV-negative subgroups of non-FIP cats (G1_Pos vs. G1_Neg), and 66 overlapped. Each of these sets of genes was further split by direction of their dysregulation (e.g., genes DE in the MLN of FIP cats but not in FCoV-positive cats non-FIP cats, and associated with a positive expression log2-ratio). This resulted in a low number (30) of genes upregulated in FCoV-positive non-FIP cats, and no significant functional enrichment was found. In contrast, there were over 1000 genes upregulated exclusively in the MLN from cats with FIP, these showed myriad enrichment for inflammatory features (including Myd88 toll-like receptors (TLR) signaling), and apoptosis. In looking at the individual genes upregulated in the MLN from cats with FIP and not in FCoV-positive non-FIP cats, some of note include CXCL10, TLR2, TLR4, TLR8, IL-1β, IL-6, and TNF. Genes involved in IFN pathways (e.g., ISG15 and 20, TRIM25, IFIT2, and IFIT3) are found elevated in the overlap of both FCoV-positive groups (G2 and G1_Pos). FOXP3 is amongst the 955 genes lower in FIP than in FCoV-positive non-FIP cats and not significantly DE in the latter.

### 2.7. Evaluation of GO Categories at the Gene Level

**Figure 8:**
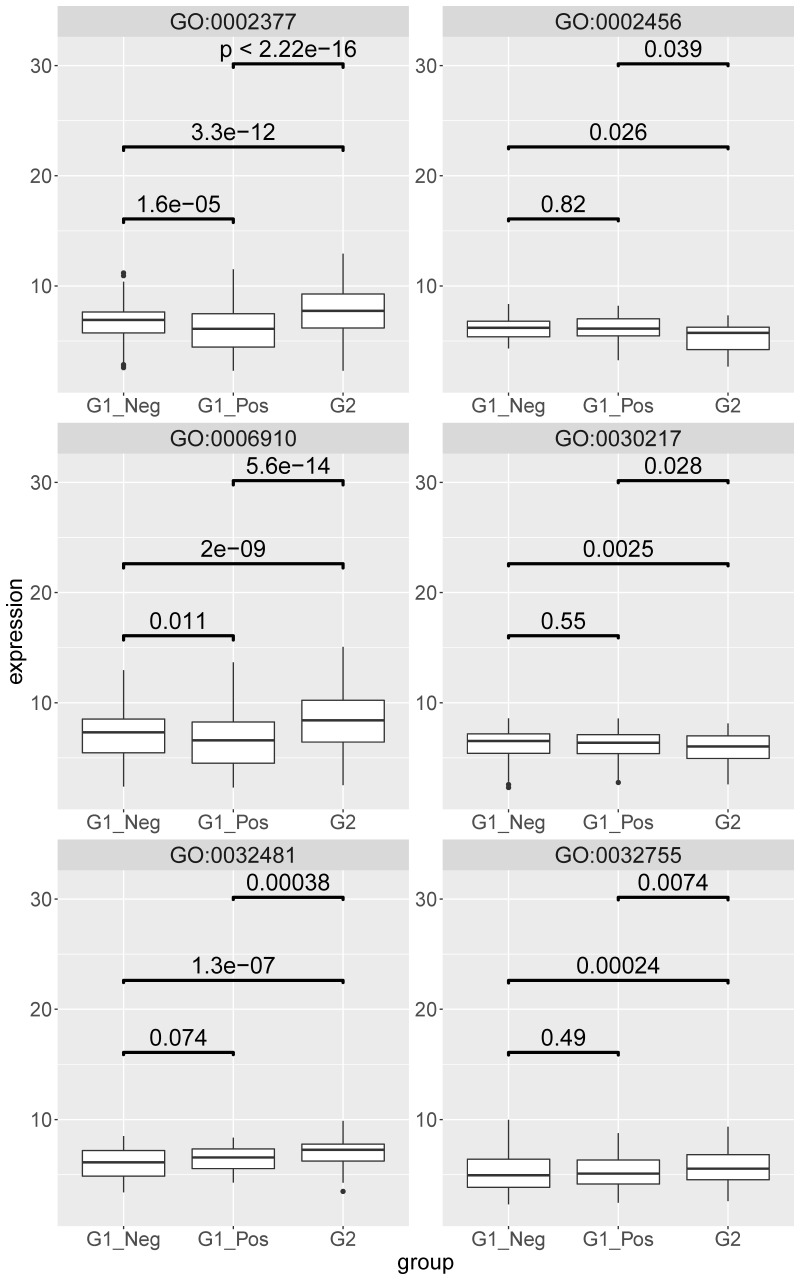
Boxplot of the mesenteric lymph node expression levels of the genes identified beyond background noise and annotated to the following gene ontology (GO) terms: GO:006910-phagocytosis recognition; GO:032481-positive regulation of type I interferon production; GO:030217-T cell differentiation; GO:002456-T cell mediated immunity; GO:032755-positive regulation of interleukin-6 production; GO:002377-Immunoglobulin production. G1_neg–non-FIP FCoV negative; G2-FIP; G1_pos–non-FIP FCoV positive. The value is the log-scaled expression from the individual genes. *p*-values are calculated with a Wilcoxon signed rank test.

A subset of GO biological processes deemed of most interest was further analyzed in order to compare ensemble gene expression within categories between groups (see Figure 8). Considering the implication of cell mediated immune response mechanisms we chose to interrogate other T cell-related pathways at the gene level, in addition to reporting the reduced role in FIP cats of the T cell receptor signaling pathway, to see if a similar pattern could be identified.

In most cases the gene expression levels were higher in the MLN from cats with FIP (G2) than in both non-FIP subgroups (G1_Pos, G1_Neg). GO:006910 (phagocytosis recognition) additionally showed significantly higher levels in FCoV-negative than in FCoV-positive non-FIP cats; within GO:0032481 (positive regulation of type I interferon production) gene levels between the two non-FIP groups were found to be slightly higher but not significantly so in FCoV-positive cats. The two T cell-related categories (GO:0030217; T cell differentiation and GO:0002456; T cell mediated immunity) both had gene levels in MLN from FIP cats significantly lower than in the non-FIP subgroups, which were themselves not significantly different from each other. Genes of GO:0032755 (positive regulation of interleukin-6 production) were higher in MLN from FIP cats than in either non-FIP subgroup, which were again similar between themselves. Immunoglobulin production category genes (GO:0002377), though higher in the MLN from FIP cats, were lower in FCoV-positive than in FCoV-negative non-FIP cats.

## 3. Discussion

The results of the present study for the first time provide detailed information on how the immune system responds to FCoV infection in natural cases, both in animals succumbing to FIP and those showing no clinical or pathological signs attributable to FIP. In focusing on the MLN, we chose to study an organ that is consistently involved in FCoV infection [50], as well as being an immune organ with a vital role in both detecting and responding to the virus. Significant differences between cats with and without FIP were observed. These were surprisingly consistent given the variation in the disease stage, distribution of lesions, and clinical findings of the FIP cases as well as the varying clinical conditions of the cats without FIP. This may reflect the fact that, despite clinically high variability, the pathogenic and pathological processes are stereotypic in FIP. In studying the MLN, we appreciate that different cell types and subsets contributed to the results, but the main cell types present will generally be B cells, T cells, and macrophages (the latter having been shown to increase in number in FIP [39]). Viral loads varied greatly between cats with FIP (up to a 100-fold) despite similar RNA-sequencing based transcriptional profiles, showing that the number of infected cells is not likely to be crucial to the organ’s response.

Thousands of genes were found to be dysregulated in the MLN of cats with FIP, pointing to numerous immune and inflammatory pathways, most of which were predictable or consistent with known features of the disease. Viral defense and negative regulation of viral replication were found to be enriched in FCoV infection regardless of FIP status, but at significantly higher levels in cats with FIP, demonstrating that the host attempts, albeit unsuccessfully in cats with FIP, to respond appropriately. Similarly, processes related to the prototypic anti-viral cytokines, type I and II IFN were also enriched in FCoV infection regardless of FIP status, whilst IFN pathway genes were heavily overrepresented amongst the top DE genes found to be higher [51]. However, type I IFN genes themselves were not upregulated. Pro-inflammatory cytokine activation was also a feature; processes upregulating all three of the feline inflammatory triad of IL-1β, IL-6, and TNF-α [52] were enriched in the MLN of cats with FIP in the present study (as were the mediators themselves when previously investigated [12]), again consistent with clinical signs. These were upregulated to a lesser degree in FCoV-positive cats without FIP, supporting a beneficial intermediate stage of activation. We also found enhanced complement pathways which complements observations of increased circulating complement in the disease state [53]. Phagocytosis was identified as an enriched process in the MLN of cats with FIP but not in FCoV-positive cats without FIP, in which it was actually lower than in FCoV-negative cats. This may be host- or virus-specific and in some way protective in FIP, linking to the phenomenon of antibody-dependent enhancement observed in experimental FIP, seemingly representing another detrimental feature of the immune response in this disease [54]. The relative lack of observed anti-inflammatory regulatory processes in the MLN of cats with FIP may help explain the exuberance of the response, despite previous studies not finding consistently excessive levels of the inflammatory cytokines themselves [12,30,33,41].

We also identified that bacterial response pathways were upregulated in the MLN of cats with FIP. There are two likely explanations for this observation. One is an overlap in function between pattern recognition receptors (PRR) responsible for detecting pathogens, e.g., TLRs 2, 4, and 8 were all upregulated in the MLN of cats with FIP and yet not in the MLN from FCoV-positive cats without FIP. This fits with previously reported findings [12]. TLR2 is classically responsible for detecting bacterial antigens but is suspected, from a human in vitro model, to have a role in detecting SARS-CoV spike proteins [55,56]. TLR4 detects the lipopolysaccharides of Gram-negative bacteria. However it appears to have a role in viral detection as mice deficient in this receptor were found to have increased susceptibility to murine hepatitis virus, a betacoronavirus [57,58,59]. Both TLR2 and 4 may also potentially react to alarmins, endogenous ligands released from damaged cells [60]. TLR8, as a ssRNA sensor [61], is the only TLR of these three which would have been predicted to be elevated in FCoV-infected cats. As none of these TLRs were elevated in the MLN of cats positive for FCoV in the absence of FIP, this may reflect that a threshold level of the virus is required to activate their production (viral levels being at least a 1000-fold different between the FCoV-positive cats with and without FIP). The range of antigen specificities across the three TLRs make it unlikely that non-pathogenic FCoV would evade receptor triggering through intrinsic viral factors. The TLRs use Myd88 signaling, as well as NF-κB [55]; the detected increases in these pathways are therefore likely to be linked. An alternative explanation for the upregulation of bacterial response pathways is that as the MLN is exposed to any intestinal pathogen, there is an increased likelihood of concurrent bacterial infection(s) stimulating PRR/TLR responses and contributing to a permissive inflammatory environment. Co-infection would certainly be an avenue for future exploration. The upregulation of collagen synthesis in the MLN of cats with FIP likely reflects the progressive fibrosis in association with multiple granulomatous infiltrates that is noted within the MLN in chronic cases of FIP (unpublished data).

There were few processes found to be significantly downregulated in the MLN of cats with FIP, but those that were have major immunological implications. These included T cell receptor signaling pathway, and IL-4 production. When genes from the categories T cell differentiation and T cell mediated immunity were analysed separately, T cell features were relatively lower in FIP cats than either group of cats without FIP. Consistent with this, anti-viral T cell responses (as determined by proliferation assays and T cell subsets) were found to be higher in cats resistant to experimental FIP in a recent study [62]. This supports a deficiency in cats that succumb to the disease, either at the level of initial detection (cell surface receptor signaling) or in sustaining an appropriate response. In the case of the latter, the FCoV-positive cats without FIP may represent a precursor stage at which the host immune system is able to contain the virus. One gene of significance here was FOXP3, upregulated in both groups of cats without FIP. FOXP3 is a transcription factor with a central role in the function of CD4^+^ regulatory T (T_reg_) cells [63]. T_reg_ cells are crucial for maintaining immune homeostasis and can also suppress other leukocytes, a function which would be expected to be beneficial in cats with an exuberant immune response [63]. A reduction in T_reg_ cells, and hence their immune regulatory effects, has also been shown in the peripheral blood of cats with FIP [64]. IL-4 is a classical Th2 cytokine, responsible for pushing Th2 differentiation and suppressing Th1. Whilst its decreased levels in the MLN of cats with FIP may appear slightly counter intuitive, given that FIP is associated with a balance skewed to Th2, this agrees with previous studies on cytokine levels and may represent end-stage disease [33,41]. It has also been found to be lower in Holy Birman cats, a breed predisposed to FIP in Italy, than in a mixed population of other breeds [65].

Results in FCoV-positive cats without FIP show that many of the same processes as in cats with FIP are significantly upregulated, as compared to FCoV-negative cats without FIP, including viral defense, leukocyte chemotaxis, IL-1 response, and IFN-response pathways. However, a closer look at the individual gene levels of these processes shows that they are higher in cats with FIP than in both groups of cats without FIP. This suggests that quantitative differences may be key. Indeed, in both FCoV-positive groups (i.e., cats with and without FIP) there was upregulation of anti-viral IFN-related genes but the inflammatory triad of IL-1, IL-6, and TNF, as well as CXCL10, a highly inflammatory chemokine, were only elevated in the MLN of cats with FIP. Therefore, these are not likely to have any positive contribution to viral defense and rather act to induce the observed pathological processes and clinical signs. Despite the upregulation of type I IFN pathways, IFN-α and-β themselves were not significantly upregulated in the MLN of cats with FIP, suggesting that further investigation into defects in this pathway may be required. Transcription factors for IFN pathways (including IFN-regulatory factors (IRF7 and 9), as well as IFN-stimulated genes (e.g., ISG15 and 20) and IFN-induced proteins (IFIT2 and 3) were all elevated in both FCoV-positive groups. Members of the large (>70 member) tripartite motif-containing (TRIM) family, some of which are involved in innate antiviral immunity [66], were upregulated in both FCoV-positive groups and are also of interest. These proteins are involved in ubiquitination, one of the effects of which is intracellular immune signal regulation. They are expressed in response to IFN and can be predicted to have both positive and negative effects on the immune response. TRIM targeting is also a virus evasion strategy and has been shown as a mechanism of SARS-CoV [67,68]. Some have intrinsic anti-viral properties, e.g., TRIM25 [66], which was upregulated in both FCoV-positive groups. However, TRIM25 can also trigger inflammatory cytokine as well as IFN production [66]. On the other hand, TRIM21, a protein that can degrade IRFs and thus reduce the IFN response [69], was only elevated in the MLN of cats with FIP. TRIM21 can also detect intracellular, antibody-opsonized virus which it targets for ubiquitination and degradation [69]. Subsequently, the viral nucleic acid is released to be detected by PRRs in the cytoplasm. So far this has only been identified as a relevant mechanism for non-enveloped viruses, however the effect of TRIM21 levels on IFN would be an interesting topic for in vitro experiments of the FCoV infection.

Crucially, there is less upregulation of humoral immunity (e.g., immunoglobulin production, B cell activation) in FCoV-positive cats without FIP in contrast to cats with FIP, further supporting the early theories that this branch of the immune system is detrimental [6,45]. In cats with FIP surviving to chronic stages of the disease, plasma cells replace macrophages in the granulomas and form layers in serositis; a morphological evidence of excessive humoral activation [28].

Previous studies have found apoptosis to be a prominent feature in FIP, both morphologically in the lymphoid tissue and at the signaling pathway level in macrophages [39,41,42]. In the current study, intrinsic apoptotic pathways were found to be enriched in the MLN from cats with FIP (though not found within the top ten categories). This may be because in end-stage FIP the active phase of lymphoid depletion had already passed.

## 4. Materials and Methods

### 4.1. Case Selection

The study population consists of animals presented to either the university small animal hospitals or local practices in Zurich, Switzerland, and Bristol, UK. All animals, with or without FIP, were euthanized for purely clinical reasons. Cases are a subset of those previously published with the exception of sample 2.9. University of Bristol FIP Biobank samples were collected at post-mortem examination with full informed consent from owners that samples could be used for research purposes. The collection, storage and use of samples used in this project were approved under ethical review by the University of Bristol Animal Welfare and Ethical Review Board (VIN/14/013; VIN/18/003) [12,13,14]. Case signalment is presented in Supplementary Table S1. Group 1 (G1) consists of 14 cases in which the diagnosis of FIP had been excluded (non-FIP cats). Following FCoV RT-qPCR on the MLN, G1 was subdivided into six G1_Pos cats (non-FIP with FCoV-positive MLN) and eight G1_Neg cats (non-FIP with FCoV-negative MLN). Group 2 (G2) consisted of ten cats with FIP. The diagnosis was based on clinical findings, macroscopical and histological alterations, together with the positive immunohistological detection of the FCoV antigen (mouse monoclonal primary antibody [clone FIPV3-70 Santa Cruz, Heidelberg, Germany]) within macrophages in typical lesions [70].

### 4.2. RNA Extraction from the MLN, cDNA Synthesis, and RT-qPCR for the Detection of FCoV

For RNA extraction, the RNeasy Plus Minikit^®^ (Qiagen) with on column genomic DNA (gDNA) removal was used following the manufacturer’s instructions. Briefly, approximately 30 mg of tissue was thawed on ice and RNA extraction buffer was added. Following disruption and homogenization using a tissue homogenizer (Mixer-Mill 300, Retsch, Haan, Germany), RNA was extracted and eluted. Remaining gDNA was removed by an additional DNase step (ezDNase; Thermo Fisher Scientific; Waltham, MA, USA). Complementary (cDNA) synthesis was performed with the High-Capacity cDNA Reverse Transcription Kit (Thermo Fisher Scientific) according to the manufacturer’s protocol. Before conducting RT-qPCR, the RNA levels of the samples were adjusted to 400 ng/μL, by measuring the concentration with a NanoDrop 2000^®^ (Thermo Fisher Scientific) and further diluted 1 in 20. TaqMan RT-qPCR was performed as previously described using a Biosystems 7500 Fast PCR System^®^ (Thermo Fisher Scientific) and published protocols [71].

### 4.3. RNA Extraction and Library Preparation for Next Generation RNA-Sequencing

A Qubit^®^ (1.0) Fluorometer (Life Technologies, Carlsbad, CA, USA) and a Bioanalyzer 2100 were used to determine the RNA quality; only samples with a 260/280 nm ratio between 1.8–2.1 and a 28S/18S ratio within 1.5–2 were processed. Following the manufacturer’s protocol, the stranded (antisense) TruSeq RNA Sample Prep Kit v2 (Illumina, Inc. San Diego, CA, USA) was used. After polyA enrichment of total RNA, the samples were reverse-transcribed into cDNA. This was followed by fragmentation, end-repair, and polyadenylation, before ligation of the TruSeq adapters (containing an index). This allows multiplexing fragments and selective enrichment by PCR. For validating the quality and quantity of the libraries, the Qubit^®^ (1.0) Fluorometer and the Caliper GX LabChip^®^ GX (Caliper Life Sciences, Inc., Waltham, MA, USA) were used. The product is a smear with an average fragment size of approximately 260 bp. The libraries were normalized to 10 nM in Tris-Cl 10 mM, pH 8.5 with 0.1% Tween 20.

### 4.4. Sequencing on NovaSeq 6000

After library quantification, libraries were prepared for loading according to the NovaSeq XP workflow with the NovaSeq6000 SP Reagent Kit (Illumina, Catalog No. 20027464, San Diego, CA, USA). Cluster generation and sequencing were performed on a NovaSeq6000 System with a run configuration of single end 100 bp.

### 4.5. Statistical Analysis

Reads were quality-checked with FastQC (Babraham bioinformatics, Cambridge, UK). Sequencing adapters were removed with Trimmomatic [72] and aligned to the reference genome and transcriptome of *Felis catus* (Ensemble, v 6.2) with STAR v 2.7.3 [73] using the following options: --outFilterType BySJout --outFilterMatchNmin 30 --outFilterMismatchNmax 10 --outFilterMismatchNoverLmax 0.05 --outMultimapperOrder Random --alignSJDBoverhangMin 1 --alignSJoverhangMin 8 --alignIntronMax 100,000 --alignMatesGapMax 100,000 --outFilterMultimapNmax 50 --chimSegmentMin 15 --chimJunctionOverhangMin 15 --chimScoreMin 15 --chimScoreSeparation 10 --outSAMstrandField intronMotif --alignEndsProtrude 3 ConcordantPair.

Distribution of the reads across genomic isoform expression was quantified using the R package *GenomicRanges* [74]. Minimum mapping quality, as well as minimum feature overlaps, was set to 10. Multi-overlaps were allowed and strand-specificity was set to 2 (antisense).

Differentially expressed (DE) genes were identified using the R package *edgeR* [75], using a generalized linear model (glm) regression, a quasi-likelihood (QL) differential expression test, and the trimmed means of M-values (TMM) normalization. In the pair-wise comparisons, genes with at least ten raw counts in at least 50% of the samples in at least one of the two groups were reported as *present* and a test was performed. Of those, genes with a *p* < 0.01 and a log2 fold-ratio > 0.5 (<−0.5) were marked as differentially upregulated (respectively downregulated).

Enrichment of GO terms was performed using the hypergeometric test as implemented in the R function *enricher,* from the package *clusterProfiler* [76].

The multi-dimensional scaling plot was generated with the R function *plotMDS* from the package *limma* [77] and the heat map with the R package *pheatmap* [78].

The potential effect of additional covariates has been tested using PERMANOVA (with 999 permutations) as implemented in the R function *adonis2* from the package *Vegan* [79].

All R packages used were from Bioconductor Version 3.10.

For all pairwise comparisons, the enrichment plots show the top 10 most enriched biological processes (GO BP) based on significantly up or downregulated genes. Tables with the full lists of significantly enriched GO terms are provided in the Supplementary Materials.

## 5. Conclusions

In conclusion, our findings highlight the difference in host response to FCoV in the context of systemic infection both in cats with and without FIP. They demonstrate the extent of immune system activation in FIP as well as the skew towards a pro-inflammatory cytokine response and further demonstrate the importance of a strong T cell-mediated immunity in mounting an effective response to FCoV infection. Comparisons of host response to infection with different clinical outcomes (i.e., FIP vs. no FIP) also support the initial hypothesis that an excessive pro-inflammatory response is responsible for FIP.

## Supplementary Materials

The following are available online at https://www.mdpi.com/2076-0817/9/7/524/s1, Figure S1: Summary of the reads mapping and gene quantification. (A) Box-plots of the distribution across the samples of the fraction (in%) of reads mapped to the genome and to coding regions (relative to the number of raw reads) and of the genes identified beyond background noise (relative to the number of genes in the annotation). (B) Number of differentially expressed genes in the pair-wise comparisons; Figure S2: Percentage of sequencing reads mapping to the rRNA silva database and showing strandedness information; Figure S3: Multidimensional scaling plot showing the main segregation of G2 from both G1 subgroups along the leading dimension. In these plots, the covariates of interest are also annotated and reveal no particular structure. The negligible effects from the covariates have also been tested using a PERMANOVA test. (A) Age: The samples are grouped by age intervals. P-PERMANOVA = 0.536. (B) Breed: The samples are grouped by breed. P-PERMANOVA = 0.734. (C) Breed: The samples are grouped by sex. P-PERMANOVA = 0.173; Table S1: Signalment and relevant pathological findings of all cases; Table S2: GO categories significantly enriched (Benjamini-Hochberg fdr < 0.05) for significantly upregulated and downregulated (italics) genes in the MLN of FIP cats compared to non-FIP cats; Table S3: GO categories significantly enriched (Benjamini-Hochberg fdr < 0.05) for significantly upregulated and downregulated (italics) genes in the MLN of FIP cats compared to FCoV-negative non-FIP cats; Table S4: GO categories significantly enriched (Benjamini-Hochberg fdr < 0.05) for significantly upregulated and downregulated (italics) genes in the MLN of FIP cats compared to FCoV-positive non-FIP cats; Table S5: GO categories significantly enriched (Benjamini-Hochberg fdr < 0.05) for significantly upregulated and downregulated (italics) genes in the MLN of FCoV-positive non-FIP cats compared to FCoV-negative non-FIP cats; Table S6: The top 30 significant differentially expressed genes in the MLN of FIP vs. non-FIP cats.
