# Supplementary material for: The Effect of Natural Feline Coronavirus Infection on the Host Immune Response: A Whole-Transcriptome Analysis of the Mesenteric Lymph Nodes in Cats with and without Feline Infectious Peritonitis
Source: Pathogens. 2020 Jun 29;9(7):524. doi: 10.3390/pathogens9070524 (PMC7400348; doi:10.3390/pathogens9070524)
Supplement: Supplementary file 1 [file pathogens-09-00524-s001.zip › new Table S1 signalment.docx]

**Table S1:** Signalment and relevant pathological findings of all cases.

| Case | Group | Signalment | Diagnosis | MLN histology | C_T_ value  (RT-qPCR) |
| --- | --- | --- | --- | --- | --- |
| 1.1 | G1_Neg | 13 y, MN, Birman | Pyothorax and pneumonia | Neutrophilic and histiocytic inflammation | ND |
| 1.2 | G1_Neg | 11 y, MN, Bengal | Colonic adenocarcinoma | Normal | ND |
| 1.3 | G1_Neg | 14 y, MN, House cat | Cerebral hemorrhage | Follicular hyalinosis | ND |
| 2.1 | G1_Neg | 8 y, FN, DSH | Chronic enteropathy | NA | ND |
| 2.2 | G1_Neg | 1 y, FN, DSH | Poxviral pneumonia | NA | ND |
| 2.3 | G1_Neg | 4 y, FN, DSH | Hepatic encephalopathy | NA | ND |
| 2.4 | G1_Neg | NA, F, DSH | Behavioral | Normal to reactive hyperplasia | ND |
| 2.5 | G1_Neg | 3 y, FN, DSH | Invasive meningioma | Normal to reactive hyperplasia | ND |
| 1.7 | G1_Pos | 1 y, FN, Maine Coon | Connective tissue abnormality and pleural effusion | NA | 39.8 |
| 1.8 | G1_Pos | 4 y, FN, Havana | Nasal lymphoma | NA | 37.6 |
| 1.9 | G1_Pos | 10 y, MN, DSH | Round cell neoplasia | Sinus histiocytosis | 36.4 |
| 2.11 | G1_Pos | 10 y, MN, DSH | Lymphoma | Normal | 38.5 |
| 2.14 | G1_Pos | 8 y, MN, DSH | Pleural effusion | Normal | 37 |
| 2.15 | G1_Pos | 18 y, FN, DSH | Chronic kidney disease | Sinus histiocytosis | 34.9 |
| 1.4 | G2 | 5 m, FN, DSH | FIP | Necrotizing and pyogranulomatous | 19.8 |
| 1.5 | G2 | 10 m, FN, Ragdoll | FIP | Necrotizing and granulomatous | 15.8 |
| 1.6 | G2 | 10 m, MN, BSH | FIP | Sinus histiocytosis | 15.1 |
| 2.6 | G2 | 4 m, F, Abyssinian | FIP | Pyogranulomatous | 17.6 |
| 2.7 | G2 | 1 y, FN, BSH | FIP | Pyogranulomatous | 17.2 |
| 2.8 | G2 | 2 y, MN, DSH | FIP | Granulomatous | 18 |
| 2.9 | G2 | 3 y, M, Oriental | FIP | Granulomatous | 19.6 |
| 2.10 | G2 | 2 y, MN, BSH | FIP | Necrotizing and pyogranulomatous | 18.1 |
| 2.12 | G2 | 12y, M, DSH | FIP | Pyogranulomatous | 20.2 |
| 2.13 | G2 | 10y, FN, DSH | FIP | Necrotizing and pyogranulomatous | 19.8 |

G1_Neg – non-FIP cats with a negative FCoV RT-qPCR result on the MLN; G1_Pos – non-FIP cats with a positive FCoV RT-qPCR result on the MLN; G2 – cats with FIP; y – years; m – months; NA – not available; F(N) – female (neutered); M(N) – male (neutered); ND– not detected; DSH – domestic short hair; BSH – British Shorthair
