# Supplementary material for: The Effect of Natural Feline Coronavirus Infection on the Host Immune Response: A Whole-Transcriptome Analysis of the Mesenteric Lymph Nodes in Cats with and without Feline Infectious Peritonitis
Source: Pathogens. 2020 Jun 29;9(7):524. doi: 10.3390/pathogens9070524 (PMC7400348; doi:10.3390/pathogens9070524)
Supplement: Supplementary file 1 [file pathogens-09-00524-s001.zip › new table S2.docx]

**Table S2:** GO categories significantly enriched (Benjamini-Hochberg fdr < 0.05) for significantly upregulated and downregulated (italics) genes in the MLN of FIP cats compared to non-FIP cats.

| **Term** | **ID** | **fdr** | **Count** | **Size** |
| --- | --- | --- | --- | --- |
| immune response | GO:0006955 | 2.44E-13 | 78 | 213 |
| inflammatory response | GO:0006954 | 6.03E-13 | 59 | 153 |
| phagocytosis, recognition | GO:0006910 | 5.49E-11 | 31 | 41 |
| complement activation, classical pathway | GO:0006958 | 1.24E-10 | 29 | 37 |
| phagocytosis, engulfment | GO:0006911 | 1.24E-10 | 32 | 45 |
| innate immune response | GO:0045087 | 1.24E-10 | 60 | 146 |
| defense response to bacterium | GO:0042742 | 1.33E-10 | 41 | 76 |
| positive regulation of B cell activation | GO:0050871 | 3.79E-10 | 30 | 42 |
| defense response to virus | GO:0051607 | 5.68E-09 | 32 | 63 |
| immunoglobulin production | GO:0002377 | 1.23E-08 | 28 | 48 |
| G protein-coupled receptor signaling pathway | GO:0007186 | 3.10E-06 | 57 | 235 |
| B cell receptor signaling pathway | GO:0050853 | 4.13E-06 | 31 | 61 |
| response to endoplasmic reticulum stress | GO:0034976 | 4.13E-06 | 26 | 53 |
| transport | GO:0006810 | 1.60E-05 | 100 | 453 |
| response to virus | GO:0009615 | 3.42E-05 | 24 | 52 |
| positive regulation of interleukin-6 production | GO:0032755 | 4.05E-05 | 16 | 31 |
| chemotaxis | GO:0006935 | 6.11E-05 | 24 | 65 |
| neutrophil chemotaxis | GO:0030593 | 4.76E-04 | 17 | 39 |
| negative regulation of viral genome replication | GO:0045071 | 5.35E-04 | 13 | 22 |
| endoplasmic reticulum unfolded protein response | GO:0030968 | 5.35E-04 | 18 | 40 |
| chemokine-mediated signaling pathway | GO:0070098 | 6.15E-04 | 18 | 48 |
| positive regulation of chemokine production | GO:0032722 | 7.85E-04 | 8 | 11 |
| endoplasmic reticulum calcium ion homeostasis | GO:0032469 | 1.40E-03 | 9 | 12 |
| cytokine-mediated signaling pathway | GO:0019221 | 1.42E-03 | 26 | 84 |
| positive regulation of interleukin-1 beta secretion | GO:0050718 | 1.52E-03 | 8 | 11 |
| ubiquitin-dependent ERAD pathway | GO:0030433 | 1.65E-03 | 23 | 59 |
| positive regulation of I-kappaB kinase/NF-kappaB signaling | GO:0043123 | 2.40E-03 | 37 | 126 |
| defense response to Gram-positive bacterium | GO:0050830 | 2.54E-03 | 13 | 28 |
| phospholipase C-activating G protein-coupled receptor signaling pathway | GO:0007200 | 3.71E-03 | 10 | 22 |
| negative regulation of endopeptidase activity | GO:0010951 | 5.82E-03 | 20 | 56 |
| cellular response to lipopolysaccharide | GO:0071222 | 8.70E-03 | 22 | 67 |
| SRP-dependent cotranslational protein targeting to membrane | GO:0006614 | 9.06E-03 | 8 | 11 |
| positive regulation of cytokine secretion | GO:0050715 | 9.56E-03 | 9 | 18 |
| proteolysis involved in cellular protein catabolic process | GO:0051603 | 9.98E-03 | 18 | 43 |
| response to lipopolysaccharide | GO:0032496 | 1.01E-02 | 25 | 85 |
| complement receptor mediated signaling pathway | GO:0002430 | 1.26E-02 | 4 | 4 |
| response to interferon-gamma | GO:0034341 | 1.73E-02 | 7 | 11 |
| defense response | GO:0006952 | 1.95E-02 | 11 | 24 |
| potassium ion import | GO:0010107 | 1.98E-02 | 6 | 9 |
| positive regulation of autophagy | GO:0010508 | 2.03E-02 | 12 | 28 |
| response to peptidoglycan | GO:0032494 | 2.16E-02 | 4 | 4 |
| zinc ion transport | GO:0006829 | 2.16E-02 | 6 | 8 |
| cellular response to interleukin-1 | GO:0071347 | 2.16E-02 | 14 | 40 |
| zinc ion transmembrane transport | GO:0071577 | 2.21E-02 | 8 | 14 |
| ER to Golgi vesicle-mediated transport | GO:0006888 | 2.21E-02 | 21 | 64 |
| cell redox homeostasis | GO:0045454 | 2.51E-02 | 19 | 53 |
| positive regulation of RNA polymerase II transcriptional preinitiation complex assembly | GO:0045899 | 3.13E-02 | 6 | 8 |
| T cell chemotaxis | GO:0010818 | 3.25E-02 | 5 | 6 |
| cell chemotaxis | GO:0060326 | 3.29E-02 | 14 | 41 |
| lymphocyte chemotaxis | GO:0048247 | 4.14E-02 | 8 | 17 |
| positive regulation of phagocytosis | GO:0050766 | 4.42E-02 | 9 | 18 |
| positive regulation of interleukin-8 production | GO:0032757 | 4.62E-02 | 8 | 16 |
| interferon-gamma-mediated signaling pathway | GO:0060333 | 4.96E-02 | 4 | 4 |
| retrograde protein transport, ER to cytosol | GO:0030970 | 5.19E-02 | 9 | 16 |
| MyD88-dependent toll-like receptor signaling pathway | GO:0002755 | 5.82E-02 | 6 | 10 |
| lipopolysaccharide-mediated signaling pathway | GO:0031663 | 5.85E-02 | 11 | 27 |
| positive regulation of NF-kappaB transcription factor activity | GO:0051092 | 5.85E-02 | 22 | 81 |
| protein secretion | GO:0009306 | 6.39E-02 | 10 | 24 |
| positive regulation of calcidiol 1-monooxygenase activity | GO:0060559 | 7.10E-02 | 3 | 3 |
| positive regulation of fever generation | GO:0031622 | 7.10E-02 | 3 | 3 |
| negative regulation of interleukin-1-mediated signaling pathway | GO:2000660 | 7.10E-02 | 3 | 3 |
| regulation of sequestering of zinc ion | GO:0061088 | 7.12E-02 | 5 | 7 |
| positive regulation of angiogenesis | GO:0045766 | 7.41E-02 | 19 | 68 |
| proteolysis | GO:0006508 | 7.59E-02 | 65 | 336 |
| negative regulation of extrinsic apoptotic signaling pathway in absence of ligand | GO:2001240 | 7.84E-02 | 8 | 19 |
| extrinsic apoptotic signaling pathway in absence of ligand | GO:0097192 | 8.51E-02 | 8 | 18 |
| Fc-gamma receptor signaling pathway | GO:0038094 | 8.51E-02 | 4 | 5 |
| antimicrobial humoral immune response mediated by antimicrobial peptide | GO:0061844 | 8.51E-02 | 3 | 3 |
| intrinsic apoptotic signaling pathway in response to endoplasmic reticulum stress | GO:0070059 | 8.60E-02 | 9 | 19 |
| positive regulation of cAMP-mediated signaling | GO:0043950 | 8.60E-02 | 4 | 5 |
| cellular response to tumor necrosis factor | GO:0071356 | 8.85E-02 | 16 | 56 |
| protein glycosylation | GO:0006486 | 8.90E-02 | 19 | 70 |
| negative regulation of cytokine production involved in inflammatory response | GO:1900016 | 8.90E-02 | 5 | 8 |
| positive regulation of calcium ion transport into cytosol | GO:0010524 | 9.93E-02 | 4 | 5 |
| positive regulation of stress-activated MAPK cascade | GO:0032874 | 9.93E-02 | 7 | 15 |
| leukocyte migration | GO:0050900 | 9.93E-02 | 10 | 27 |
| establishment or maintenance of transmembrane electrochemical gradient | GO:0010248 | 9.93E-02 | 5 | 8 |
| hyaluronan biosynthetic process | GO:0030213 | 9.93E-02 | 3 | 3 |
| acute-phase response | GO:0006953 | 9.93E-02 | 4 | 5 |
| positive regulation of defense response to virus by host | GO:0002230 | 9.93E-02 | 7 | 15 |
| positive regulation of nitric-oxide synthase biosynthetic process | GO:0051770 | 9.93E-02 | 4 | 5 |
| positive regulation of interleukin-5 production | GO:0032754 | 9.93E-02 | 4 | 6 |
| receptor-mediated endocytosis | GO:0006898 | 9.93E-02 | 17 | 60 |
| *peptidyl-tyrosine autophosphorylation* | *GO:0038083* | *0.036738* | *14* | *38* |
