# Supplementary material for: The Effect of Natural Feline Coronavirus Infection on the Host Immune Response: A Whole-Transcriptome Analysis of the Mesenteric Lymph Nodes in Cats with and without Feline Infectious Peritonitis
Source: Pathogens. 2020 Jun 29;9(7):524. doi: 10.3390/pathogens9070524 (PMC7400348; doi:10.3390/pathogens9070524)
Supplement: Supplementary file 1 [file pathogens-09-00524-s001.zip › new Table S3.docx]

**Table S3:** GO categories significantly enriched (Benjamini-Hochberg fdr < 0.05) for significantly upregulated and downregulated (italics) genes in the MLN of FIP cats compared to FCoV-negative non-FIP cats.

| **Term** | **ID** | **fdr** | **Count** | **Size** |
| --- | --- | --- | --- | --- |
| immune response | GO:0006955 | 4.80E-14 | 73 | 214 |
| inflammatory response | GO:0006954 | 3.51E-11 | 52 | 153 |
| phagocytosis, recognition | GO:0006910 | 7.00E-11 | 29 | 41 |
| defense response to virus | GO:0051607 | 8.22E-11 | 32 | 63 |
| innate immune response | GO:0045087 | 9.54E-11 | 55 | 146 |
| phagocytosis, engulfment | GO:0006911 | 9.11E-10 | 29 | 45 |
| complement activation, classical pathway | GO:0006958 | 1.98E-09 | 26 | 37 |
| immunoglobulin production | GO:0002377 | 1.98E-09 | 27 | 49 |
| defense response to bacterium | GO:0042742 | 3.05E-09 | 36 | 76 |
| positive regulation of B cell activation | GO:0050871 | 3.53E-09 | 27 | 42 |
| positive regulation of interleukin-6 production | GO:0032755 | 8.27E-07 | 17 | 31 |
| G protein-coupled receptor signaling pathway | GO:0007186 | 4.50E-06 | 53 | 238 |
| B cell receptor signaling pathway | GO:0050853 | 7.63E-06 | 28 | 61 |
| response to endoplasmic reticulum stress | GO:0034976 | 1.48E-05 | 23 | 53 |
| response to virus | GO:0009615 | 3.21E-05 | 22 | 52 |
| endoplasmic reticulum unfolded protein response | GO:0030968 | 2.86E-04 | 17 | 40 |
| positive regulation of chemokine production | GO:0032722 | 3.48E-04 | 8 | 11 |
| transport | GO:0006810 | 3.67E-04 | 85 | 455 |
| endoplasmic reticulum calcium ion homeostasis | GO:0032469 | 3.67E-04 | 9 | 12 |
| negative regulation of viral genome replication | GO:0045071 | 5.44E-04 | 12 | 22 |
| cytokine-mediated signaling pathway | GO:0019221 | 5.99E-04 | 25 | 86 |
| cellular response to lipopolysaccharide | GO:0071222 | 8.21E-04 | 22 | 67 |
| chemotaxis | GO:0006935 | 1.47E-03 | 20 | 65 |
| collagen fibril organization | GO:0030199 | 1.78E-03 | 12 | 25 |
| retrograde protein transport, ER to cytosol | GO:0030970 | 2.88E-03 | 10 | 16 |
| positive regulation of phagocytosis | GO:0050766 | 2.93E-03 | 10 | 18 |
| positive regulation of interleukin-8 production | GO:0032757 | 3.07E-03 | 9 | 16 |
| defense response to Gram-positive bacterium | GO:0050830 | 3.07E-03 | 12 | 28 |
| positive regulation of interleukin-1 beta secretion | GO:0050718 | 6.80E-03 | 7 | 11 |
| response to interferon-gamma | GO:0034341 | 7.60E-03 | 7 | 11 |
| chemokine-mediated signaling pathway | GO:0070098 | 8.24E-03 | 15 | 48 |
| negative regulation of endopeptidase activity | GO:0010951 | 8.81E-03 | 18 | 57 |
| complement receptor mediated signaling pathway | GO:0002430 | 9.78E-03 | 4 | 4 |
| cell redox homeostasis | GO:0045454 | 9.82E-03 | 18 | 53 |
| proteolysis | GO:0006508 | 1.03E-02 | 62 | 336 |
| positive regulation of NF-kappaB transcription factor activity | GO:0051092 | 1.03E-02 | 22 | 81 |
| positive regulation of monocyte chemotaxis | GO:0090026 | 1.12E-02 | 7 | 12 |
| transmembrane transport | GO:0055085 | 1.12E-02 | 49 | 268 |
| response to peptidoglycan | GO:0032494 | 1.39E-02 | 4 | 4 |
| T cell chemotaxis | GO:0010818 | 1.61E-02 | 5 | 6 |
| lipopolysaccharide-mediated signaling pathway | GO:0031663 | 1.68E-02 | 11 | 27 |
| response to lipopolysaccharide | GO:0032496 | 1.68E-02 | 22 | 85 |
| positive regulation of ERK1 and ERK2 cascade | GO:0070374 | 1.68E-02 | 26 | 108 |
| positive regulation of autophagy | GO:0010508 | 1.98E-02 | 11 | 28 |
| collagen biosynthetic process | GO:0032964 | 2.01E-02 | 4 | 4 |
| positive regulation of cytokine secretion | GO:0050715 | 2.01E-02 | 8 | 18 |
| protein glycosylation | GO:0006486 | 2.01E-02 | 19 | 70 |
| positive regulation of I-kappaB kinase/NF-kappaB signaling | GO:0043123 | 2.19E-02 | 30 | 126 |
| carbohydrate metabolic process | GO:0005975 | 2.24E-02 | 25 | 106 |
| MyD88-dependent toll-like receptor signaling pathway | GO:0002755 | 2.57E-02 | 6 | 10 |
| Fc-gamma receptor signaling pathway | GO:0032760 | 2.62E-02 | 11 | 29 |
| interferon-gamma-mediated signaling pathway | GO:0060333 | 2.72E-02 | 4 | 4 |
| intrinsic apoptotic signaling pathway in response to endoplasmic reticulum stress | GO:0070059 | 2.74E-02 | 9 | 19 |
| positive regulation of cytosolic calcium ion concentration | GO:0007204 | 2.80E-02 | 11 | 33 |
| proteolysis involved in cellular protein catabolic process | GO:0051603 | 3.04E-02 | 15 | 43 |
| response to cytokine | GO:0034097 | 4.21E-02 | 12 | 34 |
| superoxide anion generation | GO:0042554 | 5.07E-02 | 5 | 7 |
| Fc-gamma receptor signaling pathway | GO:0038094 | 5.25E-02 | 4 | 5 |
| positive regulation of cAMP-mediated signaling | GO:0043950 | 5.25E-02 | 4 | 5 |
| negative regulation of cytokine production involved in inflammatory response | GO:1900016 | 5.25E-02 | 5 | 8 |
| cellular response to tumor necrosis factor | GO:0071356 | 5.25E-02 | 15 | 55 |
| phospholipase C-activating G protein-coupled receptor signaling pathway | GO:0007200 | 5.25E-02 | 8 | 23 |
| positive regulation of calcidiol 1-monooxygenase activity | GO:0060559 | 5.25E-02 | 3 | 3 |
| positive regulation of fever generation | GO:0031622 | 5.25E-02 | 3 | 3 |
| negative regulation of interleukin-1-mediated signaling pathway | GO:2000660 | 5.25E-02 | 3 | 3 |
| positive regulation of vitamin D 24-hydroxylase activity | GO:0010980 | 5.25E-02 | 3 | 3 |
| cellular response to interleukin-1 | GO:0071347 | 5.25E-02 | 12 | 40 |
| positive regulation of calcium ion transport into cytosol | GO:0010524 | 5.36E-02 | 4 | 5 |
| acute-phase response | GO:0006953 | 5.48E-02 | 4 | 5 |
| ubiquitin-dependent ERAD pathway | GO:0030433 | 5.64E-02 | 17 | 59 |
| protein retention in ER lumen | GO:0006621 | 6.38E-02 | 4 | 5 |
| zinc ion transport | GO:0006829 | 6.57E-02 | 5 | 8 |
| activation of innate immune response | GO:0002218 | 6.74E-02 | 5 | 8 |
| potassium ion import | GO:0010107 | 6.74E-02 | 5 | 9 |
| cell chemotaxis | GO:0060326 | 6.91E-02 | 12 | 41 |
| positive regulation of interleukin-5 production | GO:0032754 | 6.91E-02 | 4 | 6 |
| lymphocyte chemotaxis | GO:0048247 | 6.91E-02 | 7 | 17 |
| positive regulation of heterotypic cell-cell adhesion | GO:0034116 | 6.91E-02 | 5 | 9 |
| positive regulation of angiogenesis | GO:0045766 | 6.91E-02 | 17 | 68 |
| response to unfolded protein | GO:0006986 | 6.91E-02 | 5 | 8 |
| interleukin-1 beta production | GO:0032611 | 6.91E-02 | 3 | 3 |
| negative regulation of extrinsic apoptotic signaling pathway via death domain receptors | GO:1902042 | 6.91E-02 | 8 | 20 |
| T cell differentiation involved in immune response | GO:0002292 | 6.99E-02 | 3 | 3 |
| dehydroascorbic acid transport | GO:0070837 | 7.87E-02 | 3 | 3 |
| ER to Golgi vesicle-mediated transport | GO:0006888 | 7.87E-02 | 17 | 64 |
| regulation of cysteine-type endopeptidase activity involved in apoptotic process | GO:0043281 | 8.92E-02 | 4 | 6 |
| negative regulation of interleukin-1 secretion | GO:0050711 | 9.14E-02 | 3 | 3 |
| positive regulation of apoptotic process involved in mammary gland involution | GO:0060058 | 9.16E-02 | 3 | 3 |
| positive regulation of type I interferon production | GO:0032481 | 9.77E-02 | 6 | 12 |
| protein processing | GO:0016485 | 9.77E-02 | 12 | 41 |
| *receptor-mediated endocytosis* | *GO:0006898* | *7.45E-02* | *17* | *61* |
| *T cell receptor signaling pathway* | *GO:0050852* | *7.45E-02* | *12* | *38* |
| *positive regulation of interleukin-4 production* | *GO:0032753* | *7.45E-02* | *7* | *13* |
| *determination of adult lifespan* | *GO:0008340* | *8.06E-02* | *5* | *7* |
