# Supplementary material for: The Effect of Natural Feline Coronavirus Infection on the Host Immune Response: A Whole-Transcriptome Analysis of the Mesenteric Lymph Nodes in Cats with and without Feline Infectious Peritonitis
Source: Pathogens. 2020 Jun 29;9(7):524. doi: 10.3390/pathogens9070524 (PMC7400348; doi:10.3390/pathogens9070524)
Supplement: Supplementary file 1 [file pathogens-09-00524-s001.zip › new Table S4.docx]

**Table S4:** GO categories significantly enriched (Benjamini-Hochberg fdr < 0.05) for significantly upregulated and downregulated (italics) genes in the MLN of FIP cats compared to FCoV-positive non-FIP cats.

| **Term** | **ID** | **fdr** | **Count** | **Size** |
| --- | --- | --- | --- | --- |
| inflammatory response | GO:0006954 | 1.25E-11 | 47 | 154 |
| immune response | GO:0006955 | 2.06E-10 | 58 | 213 |
| defense response to virus | GO:0051607 | 4.77E-08 | 27 | 63 |
| innate immune response | GO:0045087 | 3.46E-07 | 45 | 146 |
| neutrophil chemotaxis | GO:0030593 | 5.54E-06 | 17 | 39 |
| defense response to bacterium | GO:0042742 | 8.35E-06 | 29 | 76 |
| G protein-coupled receptor signaling pathway | GO:0007186 | 8.35E-06 | 43 | 244 |
| phagocytosis, recognition | GO:0006910 | 9.57E-06 | 22 | 41 |
| phospholipase C-activating G protein-coupled receptor signaling pathway | GO:0007200 | 1.20E-05 | 11 | 23 |
| positive regulation of B cell activation | GO:0050871 | 5.19E-05 | 21 | 42 |
| complement activation, classical pathway | GO:0006958 | 5.88E-05 | 20 | 37 |
| chemotaxis | GO:0006935 | 1.86E-04 | 19 | 66 |
| phagocytosis, engulfment | GO:0006911 | 1.86E-04 | 21 | 45 |
| response to endoplasmic reticulum stress | GO:0034976 | 1.86E-04 | 20 | 53 |
| chemokine-mediated signaling pathway | GO:0070098 | 3.86E-04 | 15 | 49 |
| cytokine-mediated signaling pathway | GO:0019221 | 1.24E-03 | 21 | 83 |
| response to virus | GO:0009615 | 1.43E-03 | 18 | 52 |
| SRP-dependent cotranslational protein targeting to membrane | GO:0006614 | 1.87E-03 | 8 | 11 |
| immunoglobulin production | GO:0002377 | 3.01E-03 | 17 | 47 |
| ubiquitin-dependent ERAD pathway | GO:0030433 | 3.32E-03 | 19 | 59 |
| cell chemotaxis | GO:0060326 | 5.11E-03 | 13 | 41 |
| proteolysis involved in cellular protein catabolic process | GO:0051603 | 5.11E-03 | 16 | 43 |
| potassium ion import | GO:0010107 | 5.34E-03 | 6 | 10 |
| positive regulation of cytokine secretion | GO:0050715 | 6.55E-03 | 8 | 19 |
| negative regulation of viral genome replication | GO:0045071 | 7.10E-03 | 10 | 22 |
| B cell receptor signaling pathway | GO:0050853 | 7.10E-03 | 21 | 61 |
| complement receptor mediated signaling pathway | GO:0002430 | 9.43E-03 | 4 | 5 |
| positive regulation of RNA polymerase II transcriptional preinitiation complex assembly | GO:0045899 | 9.53E-03 | 6 | 8 |
| positive regulation of chemokine production | GO:0032722 | 1.06E-02 | 6 | 11 |
| endoplasmic reticulum unfolded protein response | GO:0030968 | 1.45E-02 | 13 | 40 |
| positive regulation of I-kappaB kinase/NF-kappaB signaling | GO:0043123 | 1.46E-02 | 28 | 127 |
| complement activation | GO:0006956 | 1.98E-02 | 5 | 6 |
| interferon-gamma-mediated signaling pathway | GO:0060333 | 1.98E-02 | 4 | 4 |
| negative regulation of interleukin-1-mediated signaling pathway | GO:2000660 | 2.64E-02 | 3 | 3 |
| positive regulation of interleukin-6 production | GO:0032755 | 2.64E-02 | 10 | 33 |
| positive regulation of autophagy | GO:0010508 | 2.64E-02 | 10 | 28 |
| lipopolysaccharide-mediated signaling pathway | GO:0031663 | 2.64E-02 | 10 | 27 |
| positive regulation of interleukin-1 beta secretion | GO:0050718 | 2.64E-02 | 6 | 12 |
| defense response | GO:0006952 | 2.68E-02 | 9 | 24 |
| protein secretion | GO:0009306 | 3.20E-02 | 9 | 24 |
| Fc-gamma receptor signaling pathway | GO:0038094 | 3.30E-02 | 4 | 5 |
| negative regulation of endopeptidase activity | GO:0010951 | 3.82E-02 | 15 | 59 |
| transport | GO:0006810 | 4.30E-02 | 64 | 462 |
| positive regulation of nitric-oxide synthase biosynthetic process | GO:0051770 | 5.01E-02 | 4 | 5 |
| zinc ion transport | GO:0006829 | 5.47E-02 | 5 | 8 |
| interleukin-1 beta production | GO:0032611 | 5.51E-02 | 3 | 3 |
| positive regulation of proteasomal protein catabolic process | GO:1901800 | 5.75E-02 | 6 | 11 |
| activation of innate immune response | GO:0002218 | 5.79E-02 | 5 | 8 |
| response to unfolded protein | GO:0006986 | 5.80E-02 | 5 | 8 |
| positive regulation of vascular endothelial growth factor production | GO:0010575 | 5.80E-02 | 7 | 20 |
| xenobiotic metabolic process | GO:0006805 | 5.81E-02 | 5 | 9 |
| cellular response to lipopolysaccharide | GO:0071222 | 5.84E-02 | 16 | 68 |
| ganglioside catabolic process | GO:0006689 | 7.05E-02 | 4 | 5 |
| ER to Golgi vesicle-mediated transport | GO:0006888 | 8.26E-02 | 16 | 64 |
| signal transduction | GO:0007165 | 8.40E-02 | 75 | 614 |
| positive regulation of type I interferon production | GO:0032481 | 8.40E-02 | 6 | 12 |
| leukocyte migration involved in inflammatory response | GO:0002523 | 8.40E-02 | 4 | 7 |
| proteolysis | GO:0006508 | 8.40E-02 | 49 | 343 |
| negative regulation of protein processing | GO:0010955 | 8.40E-02 | 4 | 6 |
| protein citrullination | GO:0018101 | 8.50E-02 | 3 | 4 |
| cellular response to interleukin-1 | GO:0071347 | 8.50E-02 | 10 | 39 |
| positive regulation of NF-kappaB transcription factor activity | GO:0051092 | 8.50E-02 | 17 | 82 |
| vasoconstriction | GO:0042310 | 8.50E-02 | 4 | 8 |
| positive regulation of interleukin-23 production | GO:0032747 | 8.50E-02 | 3 | 3 |
| cellular response to lipoteichoic acid | GO:0071223 | 8.50E-02 | 4 | 6 |
| monocyte chemotaxis | GO:0002548 | 9.16E-02 | 7 | 21 |
| *lipid metabolic process* | *GO:0006629* | *3.26E-02* | *24* | *116* |
| *peptidyl-tyrosine autophosphorylation* | *GO:0038083* | *9.24E-02* | *11* | *38* |
| *transmembrane receptor protein tyrosine kinase signaling pathway* | *GO:0007169* | *9.24E-02* | *18* | *82* |
