# Supplementary material for: The Effect of Natural Feline Coronavirus Infection on the Host Immune Response: A Whole-Transcriptome Analysis of the Mesenteric Lymph Nodes in Cats with and without Feline Infectious Peritonitis
Source: Pathogens. 2020 Jun 29;9(7):524. doi: 10.3390/pathogens9070524 (PMC7400348; doi:10.3390/pathogens9070524)
Supplement: Supplementary file 1 [file pathogens-09-00524-s001.zip › new Table S5.docx]

**Table S5:** GO categories significantly enriched (Benjamini-Hochberg fdr < 0.05) for significantly upregulated genes in the MLN of FCoV-positive non-FIP cats compared to FCoV-negative non-FIP cats.

| **Term** | **ID** | **fdr** | **Count** | **Size** |
| --- | --- | --- | --- | --- |
| response to virus | GO:0009615 | 0 | 10 | 52 |
| defense response to virus | GO:0051607 | 4.91E-09 | 10 | 63 |
| negative regulation of viral genome replication | GO:0045071 | 2.95E-04 | 5 | 21 |
| ISG15-protein conjugation | GO:0032020 | 5.45E-03 | 3 | 6 |
| lymphocyte chemotaxis | GO:0048247 | 8.86E-03 | 4 | 15 |
| monocyte chemotaxis | GO:0002548 | 1.80E-02 | 4 | 19 |
| cellular response to interleukin-1 | GO:0071347 | 1.80E-02 | 5 | 38 |
| collagen fibril organization | GO:0030199 | 2.72E-02 | 4 | 26 |
| inflammatory response | GO:0006954 | 3.37E-02 | 8 | 151 |
| positive regulation of MDA-5 signaling pathway | GO:1900245 | 3.86E-02 | 2 | 3 |
| cellular response to interferon-gamma | GO:0071346 | 4.98E-02 | 4 | 29 |
| modification-dependent protein catabolic process | GO:0019941 | 6.40E-02 | 2 | 4 |
| response to stilbenoid | GO:0035634 | 8.07E-02 | 2 | 4 |
| positive regulation of RIG-I signaling pathway | GO:1900246 | 9.12E-02 | 2 | 5 |
| neutrophil chemotaxis | GO:0030593 | 9.89E-02 | 4 | 37 |
