# Supplementary material for: The Effect of Natural Feline Coronavirus Infection on the Host Immune Response: A Whole-Transcriptome Analysis of the Mesenteric Lymph Nodes in Cats with and without Feline Infectious Peritonitis
Source: Pathogens. 2020 Jun 29;9(7):524. doi: 10.3390/pathogens9070524 (PMC7400348; doi:10.3390/pathogens9070524)
Supplement: Supplementary file 1 [file pathogens-09-00524-s001.zip › SuppFig_1.pdf]

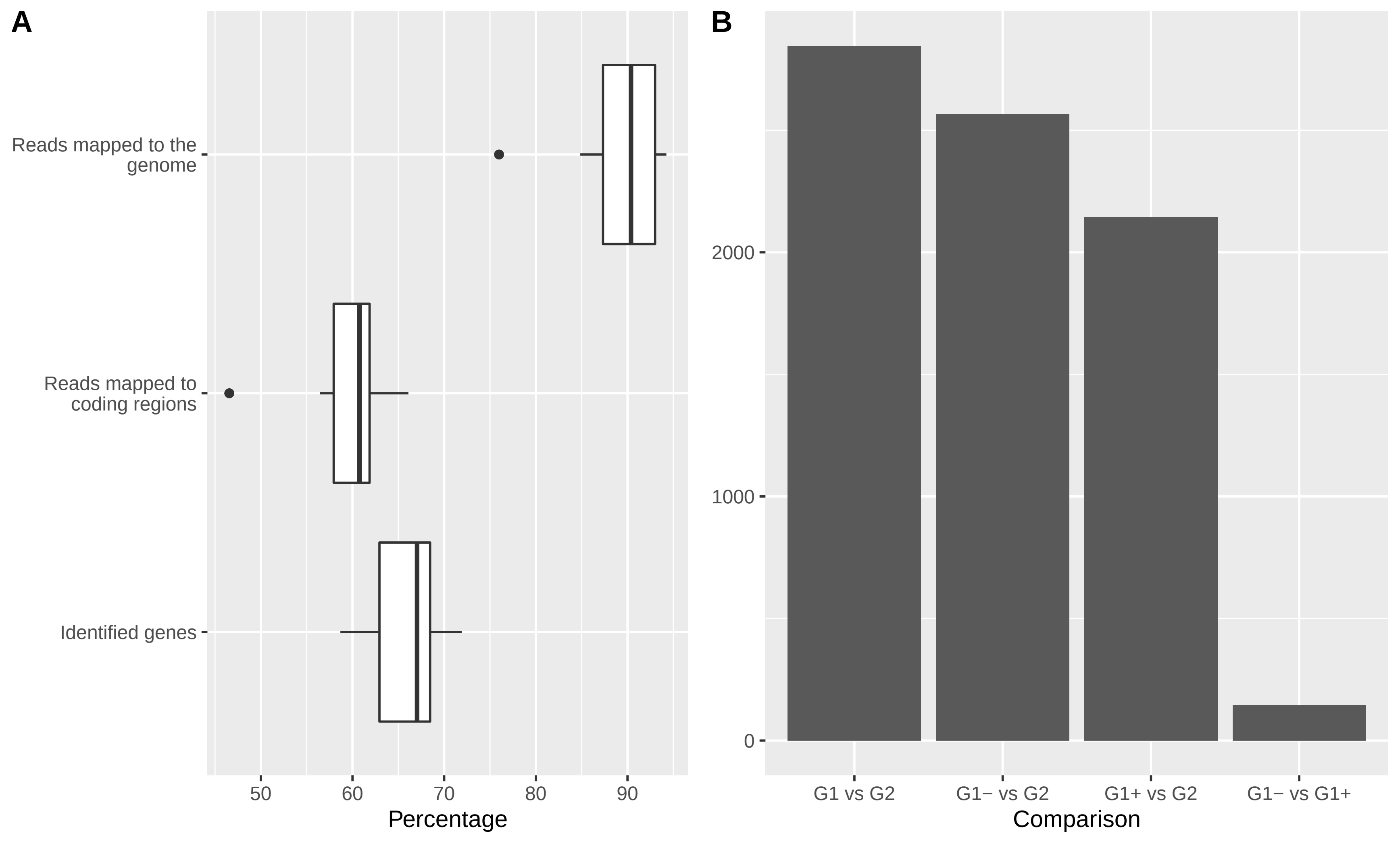

A
Reads mapped to the genome
Reads mapped to coding regions
Identified genes
50
60
70
80
90
Percentage
B
2000
1000
0
G1 vs G2
G1− vs G2
G1+ vs G2
G1− vs G1+
Comparison
