# Supplementary figures and images for: The Effect of Natural Feline Coronavirus Infection on the Host Immune Response: A Whole-Transcriptome Analysis of the Mesenteric Lymph Nodes in Cats with and without Feline Infectious Peritonitis
Source: Pathogens. 2020 Jun 29;9(7):524. doi: 10.3390/pathogens9070524 (PMC7400348; doi:10.3390/pathogens9070524)

# rRNA Silva Mapping - Bowtie2, end-to-end Alignment

antisense  
sense

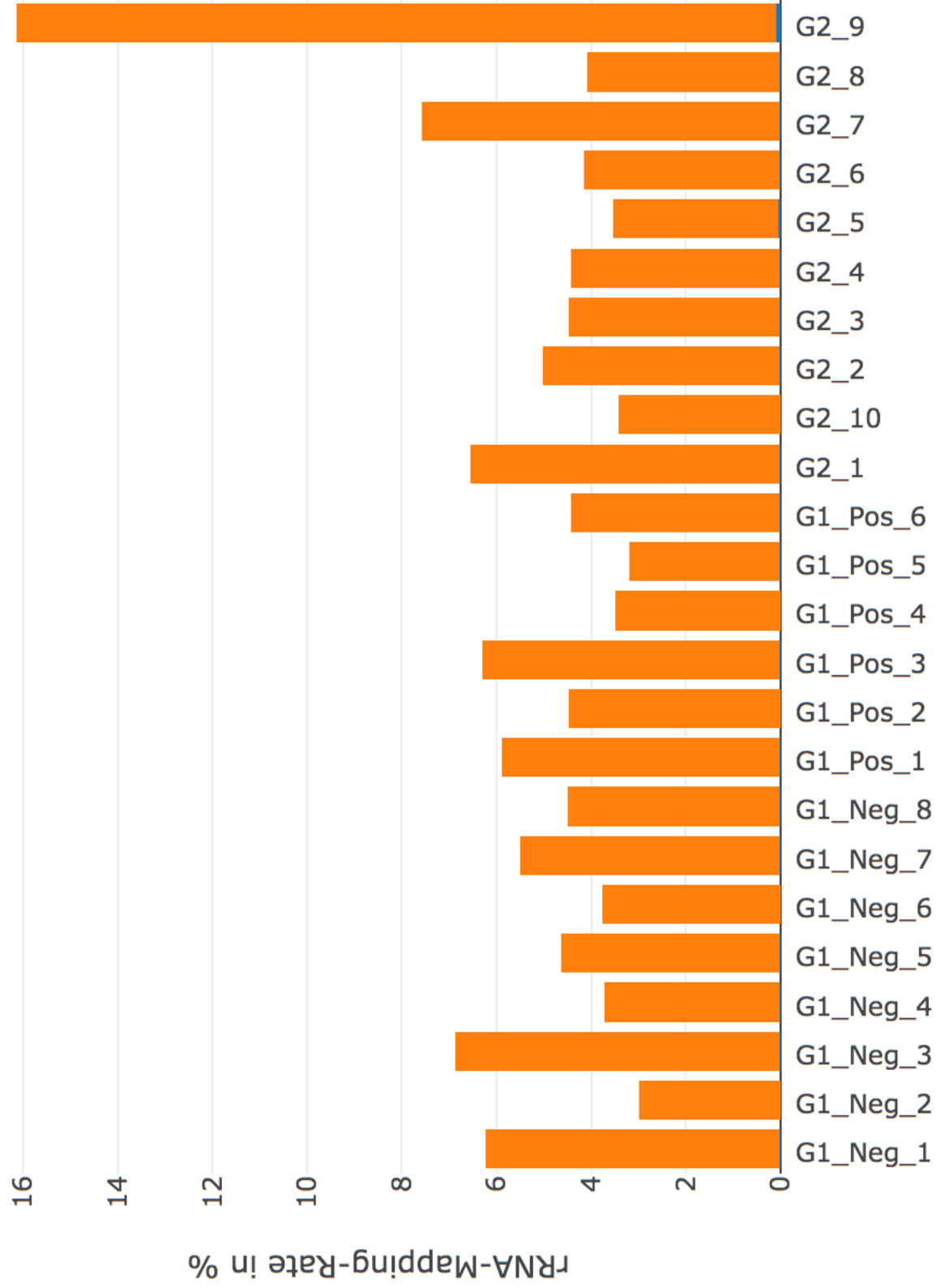

Supplement: Supplementary file 1 [file pathogens-09-00524-s001.zip › SuppFig_2.pdf]

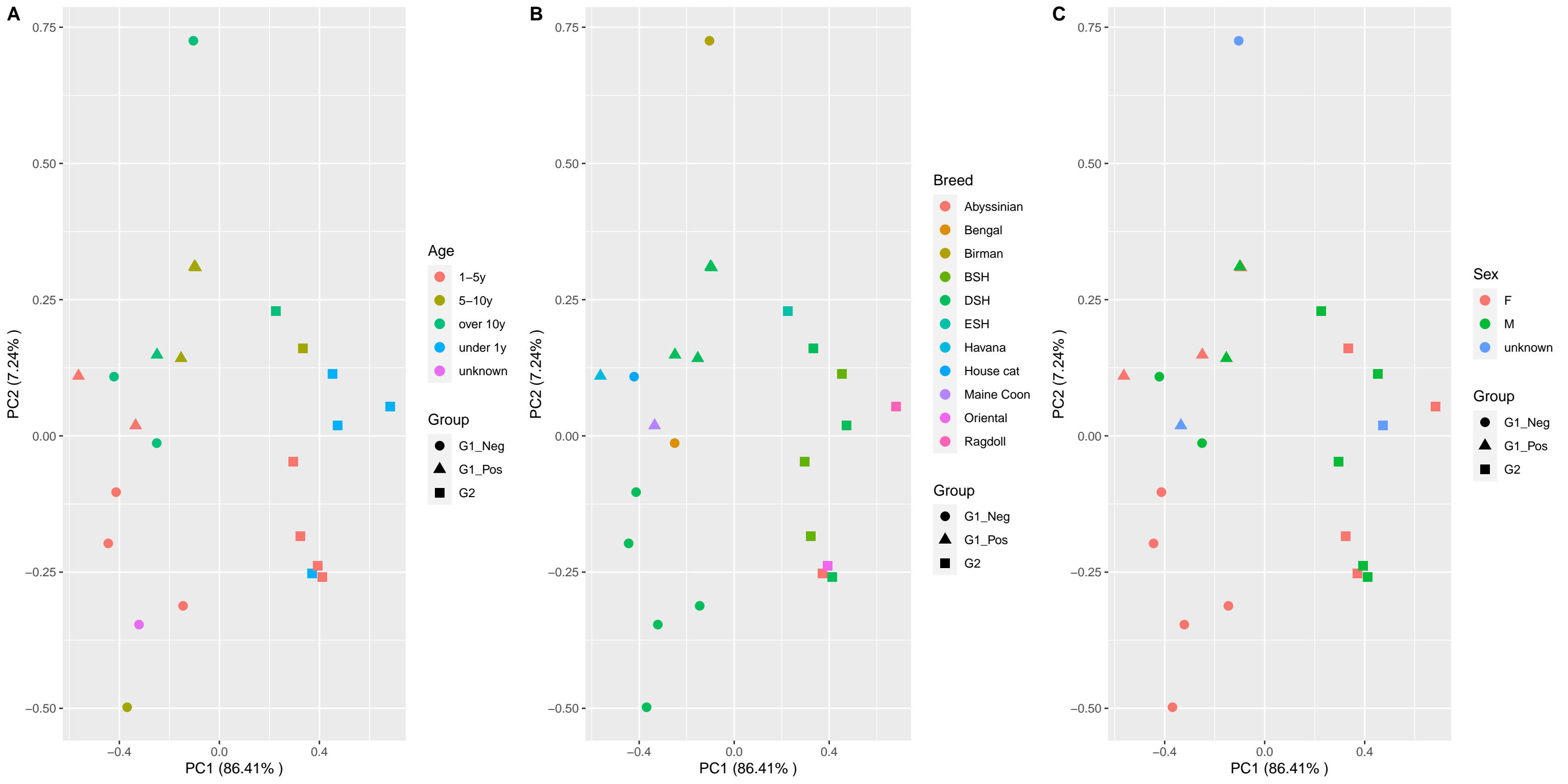

Supplement: Supplementary file 1 [file pathogens-09-00524-s001.zip › SuppFig_3.pdf]
